# *Wasabia koreana* Nakai: A Preliminary Study on Nutrients and Chemical Compounds That May Impact Sensory Properties

**DOI:** 10.3390/molecules23102512

**Published:** 2018-09-30

**Authors:** Da-Som Kim, Hoe Sung Kim, Jookyeong Lee, Jeong Hoon Pan, Young Jun Kim, Jae Kyeom Kim, Seongmin Woo, Eui-Cheol Shin

**Affiliations:** 1 Department of Food Science, Gyeongnam National University of Science and Technology, Jinju 52725, Korea; kim94dasom@naver.com (D.-S.K.); ghltjd102@naver.com (H.S.K.); tracylee0911@gmail.com (J.L.); 2 School of Human Environmental Sciences, University of Arkansas, Fayetteville, AR 72701, USA; jhpan@uark.edu (J.H.P.); jkk003@uark.edu (J.K.K.); 3 Department of Food and Biotechnology, Korea University, Sejong 30019, Korea; yk46@korea.ac.kr; 4 HANJU Industry, Yongin,16954, Korea; makesky3@naver.com

**Keywords:** *Wasabia koreana* Nakai, sinigrin, allyl isothiocyanate (AITC), volatile compounds, taste

## Abstract

In this study, the nutritional, functional, and chemical measurements of sensory attributes of different parts of wasabi, namely, leaf, petiole, and rhizome, were investigated. Proximate composition analysis showed the presence of high amounts of carbohydrates in the rhizome and amino acid composition analysis confirmed high proportions of glutamic acid and aspartic acid in all three parts. While proximate composition showed low lipid content in wasabi, ω-3 fatty acids accounted for a high proportion (>44%) of the total lipids. Wasabi leaves had high vitamin C and total phenolic contents, and thus demonstrated antioxidant capacity. Allyl isothiocyanate, which gives wasabi its characteristic pungent taste, was identified by gas chromatography/mass spectrometry and an electronic nose. On an electronic tongue, wasabi leaves showed compounds associated with sourness and saltiness while the petiole had high content of compounds associated with sweetness and bitterness. This study provides basic data for the utilization of wasabi parts as food materials based on their nutritional, functional, and chemical measure of sensory attributes.

## 1. Introduction

Wasabi, a food ingredient that is generally considered as a spice, is a cruciferous perennial half-shadow plant. Wasabi grows through water cultivation or field cultivation in cold areas [1]. The wasabi rhizome is commonly used as a pungent spice in savory fresh sea foods in Asian countries. The unique pungent taste from leaves and petioles also plays an important role in its use as a spice. It has been previously reported that wasabi inhibits vitamin C oxidation, promotes carbohydrase and thiamin activities, and has antibacterial activity, confirming its designation as a healthy food [2]. The pungent taste is generated by allyl isothiocyanate (AITC), formed as a result of hydrolysis of sinigrin, a glucosinolate compound, via myrosinase [3]. Glucosinolate compounds found in cell vacuoles in plants exist separately in intracellular organelles that are similar to vacuoles and produce diverse compounds upon hydrolysis catalyzed by enzymes (myrosinase) that are bound to the cell membranes during cell destructions [4]. Studies have reported various functional compounds in Wasabi including AITC; AITC has an improvement effect of stomach lesions of Mongolian Gerbils infected with Helicobacter pylori and affect neuroprotective and anti-inflammatory activities [5,6]. Other studies have also showed amylolytic activity in rhizome, the antimicrobial activity against human infections, and anti-hypercholesterolemic effects of wasabi in in vivo study [7,8,9]. Sinigrin and AITC contents are the highest in wasabi rhizomes and considerable amounts can be found in leaves and petioles [10]. Previous studies have reported different sinigrin and AITC contents in each part of wasabi because of the different varieties of wasabi, cultivation environment, and analysis methods. Sinigrin and isothiocyanate compounds can be extracted from wasabi parts using hot water or organic solvents such as methylene chloride, methanol, and diethyl ether. The extracted sinigrin was converted to isothiocyanate compounds, which were subjected to high-performance liquid chromatography (HPLC) and gas chromatography (GC) analysis. These results were then expressed as AITC contents [11,12]. As AITC is a volatile compound, substantial amounts of AITC can be lost while converting the extracted sinigrin to AITC at room temperature for an hour and it is therefore preferable to measure sinigrin and AITC simultaneously, rather than converting sinigrin to AITC [12].

While sinigrin and AITC have been extensively studied as the functional substances of wasabi, few studies have investigated the proximate compositions and antioxidants such as polyphenols that are present, in abundance, in plants [13]. To the best of our knowledge, the chemical measures of sensory attributes (taste and flavor) of each part of wasabi have not been studied as yet, and wasabi utilization as a spice for sea food cuisine is mostly limited to the rhizomes. This study hypothesized that besides rhizome, other parts of wasabi are also highly potential to be utilized as food materials. Therefore, the objective of this study was to investigate the nutritional, functional, and chemical measures of sensory attributes of the rhizomes, leaves, and petioles of *Wasabia koreana* Nakai to gather basic data for the development of high value-added products using different parts of wasabi.

## 2. Materials and Methods

### 2.1. Materials

*Wasabia koreana* Nakai, cultivated in water, was provided by Hanju Company (Yongin, Gyeonggie, Korea) in 2017 and the wasabi specimen (specimen voucher number: GFW-001) was stored in the specimen room of the laboratory. The wasabi specimen was separated into leaves, petioles, and rhizomes. The different parts were frozen at −40 °C before lyophilization for moisture removal. The freeze-dried samples were pulverized using a No. 20 mesh sieve for analysis.

### 2.2. Proximate Composition

Moisture, protein, lipid, and ash contents of wasabi were measured to identify proximates. Moisture content was determined from the yield after lyophilization and the crude protein, lipid, and ash contents were analyzed by the micro-Kjeldahl method, Soxhlet extraction, and direct ashing at 550 °C, based on the AOAC standards [14].

### 2.3. Constituents and Free amino Acid Composition

Acid hydrolysis method using hydrochloric acid (HCl) was used to measure the contents of constituent amino acids. Lyophilized wasabi sample (0.1 g) and 6 N HCl (3 mL) were placed in a 20 mL test tube and stirred for 10 min. The stirred sample was then pressure sealed and subjected to heat treatment in a 110 °C preheated heating block (Thermo Fisher Scientific Co., Rockford, IL, USA) for 24 h, inducing hydrolysis of the proteins to amino acids. Subsequently, HCl was removed using a rotary evaporator (R-III, BÜCHI, Postfach, Switzerland) at 50 °C. The sample was diluted to 50 mL with sodium dilution buffer and 1 mL of this solution was filtered through a 0.2 μm membrane filter. The filtered sample was then quantified by an automated amino acid analyzer (L-8900, Hitachi High Tech, Tokyo, Japan). For quantification of the free amino acids that do not constitute proteins, 1 g of the wasabi sample was stirred with 20 mL of methanol for 10 min and subsequently centrifuged at 3000 rpm for 20 min. The supernatant was concentrated under pressure and then dissolved in 25 mL of the sample dilution buffer, followed by addition of sulfosalicylic acid (20 mL) and incubation at 4 °C for an hour. The sample was subjected to centrifugation at 3000 rpm for 20 min and then filtered through a 0.2 μm membrane filter. The filtered sample was quantified by an automated amino acid analyzer (L-8900, Hitachi High Tech, Tokyo, Japan) [15].

### 2.4. Fatty Acid Composition

Fatty acid composition of the extracted wasabi lipids was measured by methyl esterification using boron trifluoride (BF_3_)-methanol. Wasabi lipids (0.1 g) were placed in a test tube and 0.5 mL of heptadecanoic acid (C17:0) (1 mg/mL hexane) was added. Next, 0.5 N NaOH-methanol (2 mL) was added and the mixture was heated at 110 °C for 10 min using the Reacti-Therm III Heating/Stirring Module (Thermo Fisher Scientific Co., Rockford, IL, USA). The mixture was cooled to room temperature and reheated after the addition of 4 mL of BF_3_-methanol to 110 °C for 1 h. After cooling, 2 mL of hexane was added and the mixture was vortexed for 1 min to collect the hexane layer for lipid analysis. GC-flame ionization detector (FID) was performed using Agilent Technologies 6890N instrument fitted with SP-2560 capillary column (100 m × 0.25 mm *i.d.*, 0.25-μm film thickness; Agilent Technologies, Santa Clara, CA, USA), and helium (2.7 mL/min) as carrier gas. Injector and detector temperatures were set to 250 °C, split ratio was 50:1, and the hydrogen and air flow rates for flame ionization were 40 mL/min and 450 mL/min at the detector, respectively. Oven temperature was initially maintained at 130 °C for 5 min. The temperature was increased by 4 °C/min and held at 240 °C for 15 min. All analyses were conducted in triplicate. Analysis results were identified by comparing retention times with fatty acid standard references [16].

### 2.5. Mineral Composition

Mineral composition was determined by microwave acid digestion (Titan MPS, Perkin Elmer Co., Waltham, MA, USA). Lyophilized wasabi (0.5 g) was weighed and 10 mL of nitric acid was added. The mixture was heated at 180 °C for 15 min and then digested at 600 W. The mixture was subsequently filtered through a 0.2 μm filter for analysis. Mineral composition of the digested sample was analyzed using an Inductively Coupled Plasma-Optical Emission Spectrometer (ICP-OES) (5300DV, Perkin Elmer Co., Waltham, MA, USA) [17].

### 2.6. Vitamin C Content

Wasabi sample was extracted using 5% meta-phosphoric acid solution and centrifuged at 3000 rpm for 15 min. The supernatant was diluted with 5% meta-phosphoric acid and used for analysis. For HPLC analysis, Agilent 1100 series (Agilent Technologies, Palo Alto, CA, USA) was used with a Discovery C_18_ column (4.6 cm × 250 mm, 5 μm, Supelco, Bellefonte, PA, USA), UV detector (254 nm), a mixture of acetonitrile, and 50 mM NH_4_H_2_PO_4_ (6:4) as a mobile phase at a flow rate of 1.0 mL/min.

### 2.7. Total Phenolic Compounds

Total phenolic content in extracted wasabi fractionation was determined by Folin-Ciocalteu’s method [18]. Lyophilized wasabi (1 mg/mL) was diluted with distilled water and 40 μL of the mixture was withdrawn. After adding 200 μL of distilled water, 200 μL of 2 N Folin-Ciocalteu’s reagent (Sigma-Aldrich Co., St. Louis, MO, USA) was added and mixed for 30 s. Next, 600 μL of 30% Na_2_CO_3_ (Sigma-Aldrich Co.), and 160 μL of distilled water were added to the mixture and reacted at 25 °C for 2 h, followed by absorbance measurement at 750 nm. For quantification of the total phenolic compounds, gallic acid in the concentration range of 0–500 μg/mL was used as a standard and treated following the same procedure as for the sample. Total phenolic compounds were calculated from the calibration curve.

### 2.8. Antioxidant Capacity

Antioxidant activity was measured by assessing the radical scavenging activity using 1,1-diphenyl-2-picrylhydrazyl (DPPH) [19]. Extracted wasabi fractions were diluted to different concentrations (200, 400, 600, and 800 μg/mL). Then, 320 μL of 0.2 mM DPPH (dissolved in 99% ethanol, Sigma-Aldrich Company) was added to 80 μL of the sample solution and the mixture was reacted at 37 °C for 30 min. Absorbance was measured at 517 nm and radical scavenging activity was measured by the equation below.

Radical scavenging activity (%) = (1 − absorbance of sample/absorbance of control) × 100(1)

Based on the radical scavenging activity, IC_50_ value, i.e., the sample content where radical scavenging activity reached to 50%, was calculated.

### 2.9. Sinigrin and AITC Content

Sinigrin and AITC contents were determined by modifying the method outlined by Tsao et al. [20]. Lyophilized wasabi (1 g) was mixed with 50% acetonitrile (100 mL) and the mixture was extracted at 100 °C for 1 h using a reflux condenser. The extracted sample was analyzed by HPLC, Agilent 1100 series. Using a UV detector, sinigrin was detected at 228 nm and AITC at 242 nm. A C_18_ Discovery column (25 cm × 4.6 mm, 5 μm, Supelco Co., Bellefonte, PA, USA) was used along with 0.025 M NH_4_OAc (pH = 6.75) (A) and acetonitrile (B) was the mobile phase, which flowed at 1 mL/min for 12 min. Mobile phase conditions were as follows: 99% A/1% B for 2 min, increased to 50% A/50% B for 0.5 s and maintained for 7.5 min, and 99% A/1% B for 2 min. 20 μL of the sample solution was injected and HPLC analysis was performed in triplicate.

### 2.10. Volatile Compounds and Sniffing Test

Volatile compounds of wasabi were collected using 100 μm polydimethylsiloxane (PDMS) coated-solid-phase microextraction (SPME) fiber (Supelco, Bellefonte, PA, USA). The sample (3 g) and the internal standard (pentadecane, 10 μg) were placed in a headspace vial (22.5 × 75 mm, PTFE/silicon septum, aluminum cap, GERSTEL, Mülheim, Germany) and injected into the SPME fiber preheated for 5 min. The sample was heated in a 60 °C heating block for 20 min and the volatile compounds were collected for 30 min via the SPME fiber. The collected volatile compounds were analyzed by GC-MS (Agilent 7890A & 5975C, Santa Clara, CA, USA) after desorption for 10 min. For analysis of volatile compounds, HP-5MS column (30 m × 0.25 mm *i.d.* × 0.25 μm film thickness) was used. Oven temperature was maintained at 40 °C for 5 min and then increased to 200 °C at a rate of 5 °C/min. The injector temperature was set to 220 °C. Helium carrier gas flowed at 1.0 mL/min and the split ratio was 1:10. Compounds separated from the total ionization chromatogram (TIC) were identified using the mass spectrum library (NIST 12), ion fragmentation pattern, and a reference [21]. Volatile compounds were quantified by converting into peak areas of the internal standard based on the peak areas of the sample.

A sniffing test was conducted with volatile compounds separated from GC/MS using an olfactory detection port with a heated mixing chamber (ODP 3, Gerstel Co., Linthicum, MD, USA) installed in the spectrometer. Considering individual olfactory differences and decreased olfactory sensitivity over time, three trained subjects participated in the sniffing test [16].

### 2.11. Electronic Nose

For analysis, 1 mL of the sample solution was placed in a headspace vial (22.5 × 75 mm, PTFE/silicon septum, aluminum cap) and the headspace was collected while stirring at 40 °C and 500 rpm for 5 min. Volatile compounds (1000 μL) were withdrawn using an automatic sampler and injected into an electronic nose (HERACLES, Alpha MOS, Toulouse, France). DB5 column (2 m × 0.18 mm) and flame ionization detectors (FID) were used. The oven temperature was held at 40 °C for 5 s, increased to 270 °C at a rate of 4 °C/s, and maintained for 30 s. The flow rate of hydrogen gas was set to 1 mL/min. Using Kovat’s index library based AroChembase (Alpha MOS); compounds corresponding to the separated peaks were identified [22].

### 2.12. Electronic Tongue

To investigate potential of wasabi extracts as a food material, wasabi extracts were analyzed using an electronic tongue sensor (ASTREE electronic tongue II, Alpha MOS, Toulouse, France). An electronic tongue module with seven sensors (Sensor array #5, Alpha MOS, Toulouse, France) was used. Two reference sensors were utilized for calibration and five sensors that measure compounds affecting taste, SRS (sour compounds), STS (salty compounds), UMS (umami compounds), SWS (sweet compounds), and BRS (bitter compounds) sensors were used for assessing the five basic tastes. Results from the two reference sensors and five taste sensors were converted to taste scores in the range of 1–12, based on the sensor responses and overall taste distribution, was examined. The sample solution (1% concentration diluted with distilled water) was prepared and filtered through filter paper (Whatman No. 1, Whatman, Kent, UK) to eliminate impurities. 100 mL of the sample solution was placed in an analysis container and tested using the sensor. All samples were analyzed seven times. The sensor was rinsed between the tests to completely remove the residues and the tastes of the previous sample. The results have been presented in a radar plot to show a relative comparison among the different tastes [23].

### 2.13. Statistical Analysis

Nutritional and functional characteristics of wasabi were presented as mean and standard deviations of the results from experiments performed in triplicate. Significance of the mean values was tested by Tukey’s multiple range test using the SAS version 9.2 (SAS Institute Inc., Cary, NC, USA) package (*p* < 0.05).

## 3. Results and Discussion

### 3.1. Proximate Composition

The proximates of wasabi parts are shown in Table 1. It was found that all wasabi parts had a high moisture content. Crude protein and crude lipid contents were significantly high in the leaves (*p* < 0.05) and crude ash contents were significantly high in the petioles (*p* < 0.05). Rhizomes had the highest content of carbohydrates, indicating that fiber was a primary component of rhizomes. The results of proximates in diverse wasabi parts can be used as basic data to establish their use as food materials.

### 3.2. Constituents and Free Amino Acid Compositions

Constituent amino acid composition of wasabi parts is summarized in Table 2. Overall, the constituent amino acids did not exhibit any distinctive differences among the wasabi parts. All parts contained more than 60% of non-essential amino acids, showing the highest glutamic acid and aspartic acid contents. The leaf, petiole, and rhizome contained similar proportions of essential amino acids with 38.51 ± 4.17%, 38.27 ± 5.02%, and 38.27 ± 5.02% of the total contents, respectively, indicating statistically no significant result (*p* > 0.05). Lysine, leucine, and phenylalanine were confirmed to be primary essential amino acids in the leaf, petiole, and rhizome. The free amino acid composition is shown in Table 3. The highest proportions of *γ*-aminobutyric acid (GABA) were identified in the leaf, petiole, and rhizome with 33.29 ± 0.97%, 33.37 ± 1.06%, and 26.82 ± 1.15%, respectively. Gamma-aminobutyric acid (GABA) is a neurotransmitter that acts on the central nervous system in mammals. It moderates excitement in the nervous system and directly controls the muscles in human beings. While GABA is an amino acid, it does not constitute proteins as it is not an α-amino acid. As GABA stimulates the hippocampus and cortex in the mammal brains, it is an important stimulant for several parts in the brain until glutamine acid synapse is established. GABA is synthesized in the nerves before synapses connect and acts as a signal moderator between neurons. It also contributes to the dispersion, transfer, differentiation, and extension of neurite and synapse formation, regulates the growth of embryonic stem cells and neural stem cells, and affects the growth of neurons through cerebral nerve stimulators. GABA contents in all three wasabi parts were quite high, indicating the potential use of wasabi as a GABA source [24,25]. In addition, the leaf and petiole had relatively high proportions of alanine (12.49 ± 0.43% and 19.55 ± 0.61%) and valine (8.81 ± 0.17% and 9.25 ± 0.31%). Wasabi rhizome showed high proportions of arginine (13.12 ± 0.47%), threonine (11.02 ± 0.32%), and valine (10.08 ± 0.33%), which were significantly more than those in the leaf and petiole (*p* < 0.05). Studies by Kato et al. and Kang et al. reported that each amino acid has its taste intensity based on the taste threshold, i.e., the minimum concentration at which individuals start to taste. While taste of foods is elicited by a combination of various compounds, composition of individual amino acids is also known to affect the taste generation [26,27]. For example, aspartic and glutamic acids had sour taste. In addition, although glutamic acid and aspartic acid are associated with a sour taste, they generate an umami taste in aqueous solutions with NaCl at pH 6 [27].

### 3.3. Fatty Acid Composition

Fatty acid composition of the wasabi parts is given in Table 4. While relatively small proportions of crude lipid were found in the leaf, petiole, and rhizome, i.e., 0.32 ± 0.01%, 0.32 ± 0.01%, and 0.32 ± 0.01%, respectively, the fatty acid composition was nutritionally superb. Linolenic acid (ω-3), oleic acid (ω-9), and palmitic acid were the main fatty acids. Relatively, the highest proportions of linolenic acid (ω-3) were found in the leaf, petiole, and rhizome with 59.57 ± 0.12%, 52.87 ± 0.14%, and 44.25 ± 0.23%, respectively, particularly showing a significantly high proportion in the leaf (*p* < 0.05). Linolenic acid (ω-3) is an essential fatty acid that needs to be ingested from foods as it is not synthesized in the body. It constitutes lipids and functions as phospholipid of cell membranes. Further, it converts to eicosapentanoic acid (EPA) and docosahexanoic acid (DHA) via elongation (binding two carbons) and desaturation (generating double bonds) [28]. Relatively high proportions of oleic acid were observed in the leaf, petiole, and rhizome with 16.33 ± 0.09%, 14.39 ± 0.04%, and 14.22 ± 0.13%, respectively, showing a significantly high proportion in the leaf (*p* < 0.05). Oleic acid, a main component of monounsaturated fatty acids, is also an essential fatty acid. It lowers LDL-cholesterol that causes arteriosclerosis and increases HDL-cholesterol that protects the liver. It is also effective in the prevention of cardiovascular diseases and aging, and shows superior anticancer effects based on antioxidant activity [28]. More than 60% of unsaturated fatty acids were found in all three parts.

### 3.4. Mineral Composition

Mineral compositions of the different wasabi parts are summarized in Table 5. Among the ten minerals investigated, potassium was present in the highest proportions in the leaf, petiole, and rhizome with 43.75 ± 4.35%, 65.02 ± 4.21%, and 57.93 ± 3.23%, respectively, showing a significantly high proportion in the petiole (*p* < 0.05). Potassium is a major electrolyte of body fluids like sodium and controls the moisture content and acid-alkali balance with sodium in the body. Sodium is distributed outside the cells while 95% of potassium exists in the cell. Potassium-sodium balance influences blood pressure and muscular contraction and relaxation in the body. It also affects both intracellular and extracellular electric potential and regulates intracellular ionic strength [29]. Relatively high proportions of calcium were found in the leaf, petiole, and rhizome (19.66 ± 1.21%, 18.99 ± 2.09%, and 16.15 ± 1.43%, respectively), but there were no significant differences between the various parts (*p* > 0.05). Calcium plays a crucial role in the muscles and controls brain cell growth [29].

### 3.5. Vitamin C, Total Phenolic Contents and Antioxidant Capacity

Vitamin C, total phenolic content, and antioxidant capacity (IC_50_) of the different wasabi parts were determined from the DPPH assay (Table 6). Vitamin C content was significantly higher in the leaf (108.09 ± 7.43 mg/g), as compared to the petiole and rhizome (*p* < 0.05). Total phenolic content was the highest in the leaf with 1275.00 ± 170.81 mg/100 g, followed by petiole and rhizome with 420.01 ± 81.70 mg/100 g and 382.22 ± 60.60 mg/100 g, respectively. Total phenolic content is closely related to antioxidant activity and phenolic compounds are known to be special components of phytochemicals. The leaf part had the lowest IC_50_ value of 7.64 ± 0.54 mg, while the IC_50_ values of petiole and rhizome were 17.24 ± 1.17 mg and 16.95 ± 0.61 mg, respectively. This result indicated that wasabi leaves have superior antioxidant properties than the petiole and rhizome, which is in congruence with the finding that the vitamin C and total phenolic contents are the highest in the leaf. Based on this result, it can be concluded that wasabi leaves have superior antioxidant activity. A previous study also reported that the wasabi leaf possesses higher total phenolic compounds and antioxidant capacity than the other parts, which confirms the results of this study [13].

### 3.6. Sinigrin and AITC Contents

Sinigrin and AITC contents of the different wasabi parts are shown in Table 7. The amounts of sinigrin in the wasabi leaf, petiole, and rhizome were 11.95 ± 0.91 mg/g, 10.42 ± 0.23 mg/g, and 38.36 ± 2.43 mg/g, respectively. These results indicated that sinigrin was present in a significantly higher amount in the rhizome (*p* < 0.05). Similarly, AITC content was the highest in the rhizome, which is in agreement with literature [10]. Sinigrin generates allyl isothiocyanate (AITC) and other volatile compounds when it chemically reacts with oxygen and thioglucosidase. AITC is the main compound responsible for the pungent taste and favor of wasabi. It exhibits antibacterial properties and can inhibit the growth of foodborne pathogens such as *E. coli, salmonella*, *O-157*, *vibrio*, and *staphylococcus* [11]. Kim et al., have previously reported that the high volatility and instability of AITC at high temperature can lead to issues with recovery and degeneration when conventional methods of extraction at high temperature are employed [30]. Therefore, these authors developed an extraction method using supercritical carbon dioxide. Further, they found that the suggested method showed a similar extraction rate to the procedure with organic solvents. Additionally, this method effectively addressed the drawbacks of poor recovery and toxicity of residual organic solvents of the conventional extraction method. In a study by Lee, significant anti-cholesterol activity of the rhizome and leaf extracts of wasabi was reported as compared to the control [9]. Generally, wasabi use relies heavily on the rhizome, even though wasabi grows in restricted areas and is somewhat expensive. In fact, wasabi cultivation is discontinued after cutting off the rhizome, exerting a limitation in rhizome commercialization. Wasabi leaf and petiole, on the other hand, can be consistently produced at a higher rate as compared to rhizome, and sinigrin and isothiocyanate compounds extracted from the leaf and petiole can be commercialized, which may be economically efficient [2].

### 3.7. Volatile Compounds and Sniffing Test

**Figure 1:**
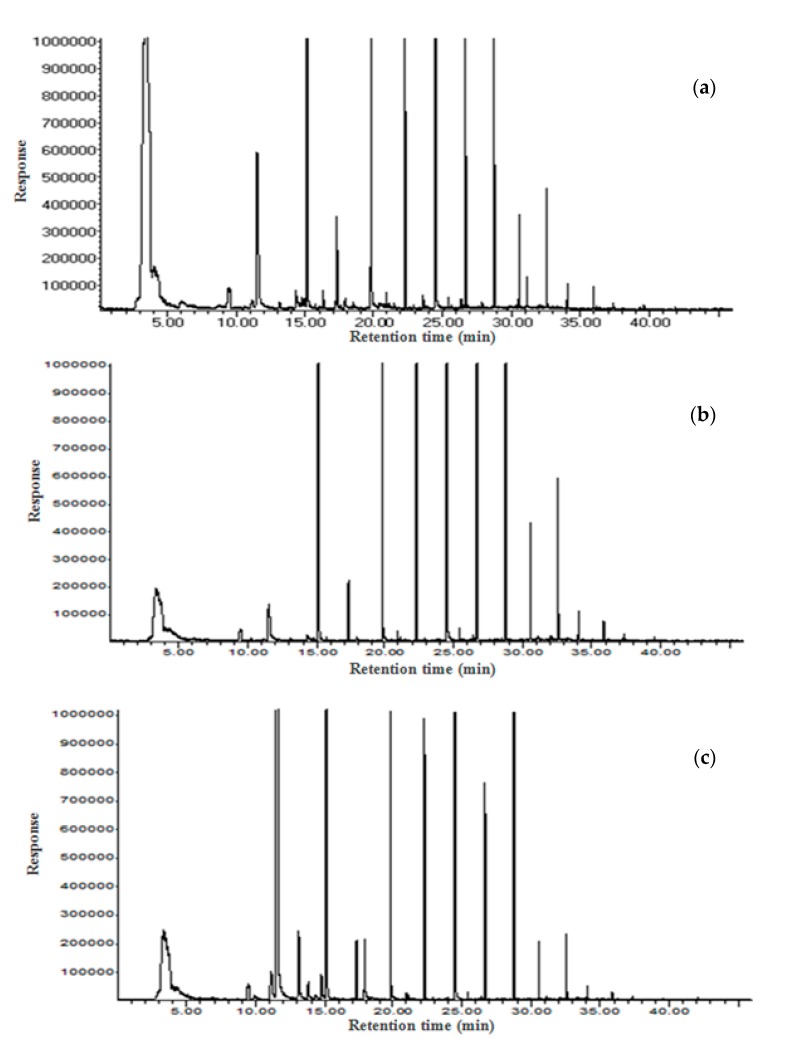
Total ion chromatograms of *Wasabia koreana* Nakai (**a**) leaf, (**b**) petiole, and (**c**) rhizome.

Volatile compounds of wasabi parts are listed in Table 8 and shown in Figure 1. A total of seventeen compounds (three acids, an alcohol, two aldehydes, four hydrocarbons, six heterocyclic compounds, and a ketone) were found in the leaf, fourteen compounds (two acids, an alcohol, two aldehydes, three hydrocarbons, and six heterocyclic compounds) were present in the petiole, and six compounds (a hydrocarbon and five heterocyclic compounds) were identified in the rhizome. AITC content was high in all three parts and the petiole had a higher content of 3-hexen-1,6-dialdehyde than AITC (9.42 ± 3.32 μg/g). AITC was identified at the retention time of 11.50 min with a retention index of 914. AITC contents in the leaf, petiole, and rhizome were 71.51 ± 8.14 μg/g, 8.99 ± 2.71 μg/g, and 117.80 ± 66.60 μg/g, respectively. This result is consistent with the AITC content determined by HPLC analysis (Table 7), showing its highest content in the rhizome followed by the leaf and petiole. In the sniffing test, volatile compounds similar to that of wasabi were identified at retention times of 11.50 min and 20.13 min. Based on the GC/MS library, the compounds were confirmed as AITC and azulene. AITC appeared as a strong peak while azulene was found only in the petiole with a weak peak. Furthermore, several sulfide compounds such as isopropyl isothiocyanate, 3-methyl isothiazole, and 4-isothiocyanato-1-butene were identified in the rhizome. In a study by Kumaga et al., thirteen isothiocyanate compounds including 3-butenyl isothiocyanate, 4-butenyl isothiocyanate, 5-hexenyl isothiocyanate, and 6-heptenyl isothiocyanate were found in wasabi produced in Japan (*Wasabia japonica* Matsum) [31]. They further reported that these compounds add a unique wasabi flavor called greenish note and *Wasabia japonica* Matsum contained 200–400 times higher amounts of these compounds than all three parts of wasabi produced in Western countries. Sultana et al. separated six isothiocyanate (ITCs) compounds in wasabi, of which AITC accounted for highest amount, followed by 4-pentenyl ITC, 3-butenyl ITC, sec-butyl ITC, isopropyl ITC, and 5-hexenyl ITC [10]. Other studies also reported the high amount of AITC [31,32]. Dai and Lim noted that AITC is proportionally increased to sinigrin generation as temperature increases [11]. Kim et al. [30] extracted isothiocyanate (ITCs) compounds including AITC using SCO_2_, [30]. They found the extraction yield similar to conventional organic solvents (ether, ethanol), and extracted a high amount of AITC from leaf and petiole by altering SCO_2_ conditions [30]. Aforementioned studies showed potentials that AITC can be extracted without using organic solvents and obtained from other parts besides rhizome.

### 3.8. Electronic Nose (E-Nose)

Volatile compounds of wasabi analyzed by the electronic nose are summarized in Table 9. It is evident from the results that wasabi AITC exhibited the highest content of all the volatile compounds. Among the ten identified peaks, the main peak was that of AITC, which showed the peak areas of 345.78 ± 48.90, 1309.28 ± 160.62, and 13,426.24 ± 653.00 in the leaf, petiole, and rhizome, respectively. AITC is characterized by its garlic, pungent, and sulfurous odor. As shown in the results in Table 8 obtained by SPME-GC/MS and in Table 9 using an E-nose, the AITC content was highest in the rhizome whereas sinigrin (Table 7) was not detected by SPME-GC/MS and E-nose as it is a non-volatile compound. Furthermore, the rhizome contained a high amount of dimethyl trisulfide, which has an odor of onion and rotten food.

### 3.9. Electronic Tongue

**Figure 2:**
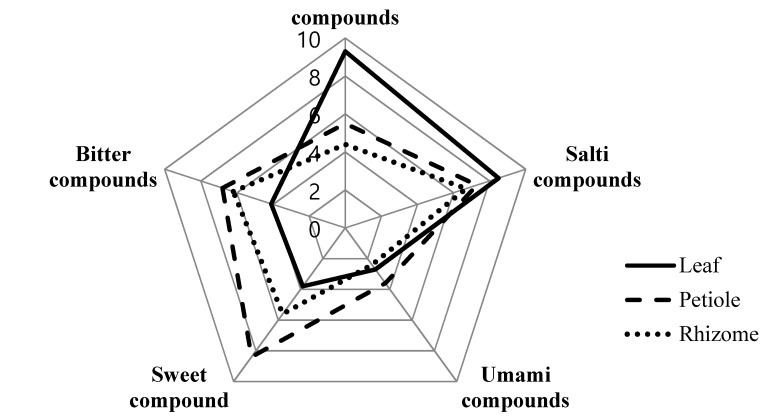
Plot of chemical sensors attributed sensory of *Wasabia koreana* Nakai parts developed using an electronic tongue.

Relative comparison of compounds associated with the five basic tastes in wasabi was analyzed by an electronic tongue (Figure 2). Compounds associated with sour and salty tastes were the most prominent in wasabi leaves. The compounds associated with umami taste did not vary as much but was the highest in the petiole. Compounds associated with sweet and bitter tastes were also the highest in the petiole. It is known from literature reports that AITC in wasabi is highly volatile, and consequently, the compound associated with pungent taste does not last long in the mouth, unlike peppers and garlic. According to Depree et al., wasabi is characterized first by its sweet taste, followed by a strong pungent taste, and a slightly bitter taste at the last, and these tastes are associated with the mechanism of pungent principle in wasabi; glucose for sweet taste, hydrogen sulfide for bitter taste, and AITC for pungent taste, which are generated in the hydrolysis of sinigrin by myrosinase [33]. Electronic nose analysis showed different results from the taste values based on amino acid profiles given in Table 2. Such difference may be attributed to the nature of complex tastes present in wasabi parts expressed by the electronic tongue sensor. The diverse tastes identified in wasabi parts can be utilized as basic data for the development of food materials based on wasabi.

## 4. Conclusions

In order to extend the scope of wasabi use, this study investigated proximate composition, functional component, and sensory attributes by chemical sensors in different parts of wasabi. All parts showed high essential amino acids content, especially ω-3 fatty acid that is beneficial to vascular health. Leaf, a relatively less frequently used part as food materials, showed higher total phenolic content and superior antioxidant capacity to the other parts, confirming high functionality of wasabi parts in not frequent use. Additionally, AITC content in rhizome has been a determinant factor of functional and sensory attributes of wasabi so far but this study confirmed AITC in leaf and petiole, a relatively small amount compared to rhizome. Such a result showed a potential of use of leaf and petiole that are possible to be reproduced rather than rhizome that is not able to be reproduced. E-tongue results confirmed varied patterns of compounds associated with tastes of leaf, petiole, and rhizome. This study provides basic data for the utilization of wasabi parts as food materials based on their nutritional, functional, and chemical measures of sensory characteristics.

## Figures and Tables

**Table 1:** Proximates in the different parts of *Wasabia koreana* Nakai.

Parts	Proximates (g/100 g)				
	Moisture	Crude Protein	Crude Lipid	Crude Ash	Carbohydrate ^(1)^
Leaf	92.50 ± 0.24 ^b(2)^	3.21 ± 0.36 ^a^	0.32 ± 0.01 ^a^	1.72 ± 0.01 ^b^	2.26 ± 0.58 ^b^
Petiole	94.37 ± 0.5 ^a^	2.45 ± 0.14 ^b^	0.13 ± 0.01 ^b^	2.07 ± 0.35 ^a^	0.98 ± 0.36 ^b^
Rhizome	81.35 ± 0.23 ^c^	2.33 ± 0.35 ^b^	0.10 ± 0.01 ^c^	0.98 ± 0.36 ^c^	15.48 ± 0.28 ^a^

Data are given as mean ± SD values from experiments performed in triplicate. ^(1)^ Total carbohydrate content of foods was calculated by difference. The other constituents in the sample such as moisture, ash, protein, and lipid were subtracted from the total weight of the sample. ^(2)^ Mean values with different letters within the same column are significantly different according to Tukey’s multiple range test (*p* < 0.05).

**Table 2:** Structural amino acid composition of the different parts of *Wasabia koreana* Nakai.

Amino Acid	Composition (%)		
	Leaf	Petiole	Rhizome
*Essential amino acids*			
Lysine	7.17 ± 1.24 ^a(^^1)^	8.51 ± 1.07 ^a^	6.15 ± 1.36 ^b^
Methionine	1.50 ± 0.12 ^a^	0.17 ± 0.05 ^b^	1.61 ± 0.09 ^a^
Leucine	7.79 ± 1.47 ^a^	6.60 ± 1.26 ^a^	4.56 ± 0.95 ^b^
Phenylalanine	7.27 ± 0.87 ^a^	6.80 ± 1.05 ^a^	5.03 ± 0.74 ^a^
Valine	4.06 ± 0.58 ^a^	4.56 ± 0.69 ^a^	5.04 ± 0.77 ^a^
Histidine	3.73 ± 0.64 ^a^	4.05 ± 0.45 ^a^	3.05 ± 0.38 ^a^
Threonine	4.96 ± 0.48 ^a^	5.19 ± 0.57 ^a^	5.57 ± 0.74 ^a^
Isoleucine	2.02 ± 0.09 ^ab^	2.39 ± 0.13 ^a^	1.79 ± 0.14 ^b^
*Non-essential amino acids*			
Arginine	7.16 ± 1.05 ^b^	4.84 ± 0.69 ^c^	10.24 ± 1.36 ^a^
Glutamic acid	18.61 ± 2.35 ^b^	23.27 ± 2.98 ^a^	20.47 ± 3.05 ^ab^
Aspartic acid	12.97 ± 3.24 ^b^	12.95 ± 3.14 ^b^	17.72 ± 2.99 ^a^
Alanine	7.33 ± 1.21 ^ab^	7.95 ± 1.36 ^a^	6.71 ± 1.45 ^b^
Glycine	6.53 ± 2.05 ^a^	6.13 ± 1.22 ^a^	4.90 ± 1.07 ^b^
Serine	5.96 ± 0.94 ^a^	6.58 ± 0.87 ^a^	5.87 ± 1.08 ^a^
Tyrosine	2.93 ± 0.15 ^a^	0.00 ± 0.00	1.29 ± 0.08 ^b^
%Essential amino acids	38.51 ± 4.17 ^a^	38.27 ± 5.02 ^a^	38.27 ± 5.02 ^a^
%Non-essential amino acids	61.49 ± 6.62 ^a^	61.73 ± 8.54 ^a^	67.20 ± 7.13 ^a^
%Total amino acids	100.00	100.00	100.00

Data are given as mean ± SD values from experiments performed in triplicate. ^(1)^ Mean values with different letters within the same row are significantly different according to Tukey’s multiple range test (*p* < 0.05).

**Table 3:** Free amino acid compositions of the different parts of *Wasabia koreana* Nakai.

Amino Acid	Composition (%)		
	Leaf	Petiole	Rhizome
Aspartic acid	1.27 ± 0.05 ^a(^^1)^	0.76 ± 0.08 ^b^	0.74 ± 0.09 ^b^
*ρ*-Serine	0.00 ± 0.00	0.33 ± 0.05	0.00 ± 0.00
Threonine	6.37 ± 0.14 ^c^	7.38 ± 0.21 ^b^	11.02 ± 0.32 ^a^
Serine	4.20 ± 0.11 ^b^	4.77 ± 0.13 ^a^	3.18 ± 0.14 ^c^
Glutamic acid	6.90 ± 0.14 ^b^	7.64 ± 0.24 ^a^	3.58 ± 0.18 ^c^
Glycine	0.38 ± 0.07 ^b^	0.41 ± 0.09 ^b^	1.70 ± 0.08 ^a^
Alanine	12.49 ± 0.43 ^b^	19.55 ± 0.61 ^a^	8.75 ± 0.25 ^c^
Valine	8.81 ± 0.17 ^b^	9.25 ± 0.31 ^b^	10.08 ± 0.33 ^a^
Cysteine	0.00 ± 0.00	0.47 ± 0.08 ^b^	0.68 ± 0.09 ^a^
Methionine	0.11 ± 0.02 ^b^	0.24 ± 0.03 ^a^	0.26 ± 0.04 ^a^
Isoleucine	3.67 ± 0.25 ^b^	3.41 ± 0.12 ^b^	3.95 ± 0.15 ^a^
Leucine	3.73 ± 0.19 ^a^	2.28 ± 0.09 ^b^	3.75 ± 0.18 ^a^
Tyrosine	2.64 ± 0.15 ^a^	1.03 ± 0.05 ^b^	2.71 ± 0.10 ^a^
Phenylalanine	5.64 ± 0.24 ^a^	2.65 ± 0.07 ^b^	2.14 ± 0.06 ^b^
*β*-alanine	2.08 ± 0.11 ^a^	1.16 ± 0.08 ^b^	1.00 ± 0.07 ^b^
*γ*-Aminobutyric acid	33.29 ± 0.97 ^a^	33.37 ± 1.06 ^a^	26.82 ± 1.15 ^b^
Lysine	1.30 ± 0.07 ^a^	0.55 ± 0.10 ^b^	0.76 ± 0.06 ^b^
Histidine	1.15 ± 0.04 ^c^	3.85 ± 0.11 ^b^	5.77 ± 0.15 ^a^
Arginine	5.97 ± 0.22 ^b^	0.90 ± 0.07 ^c^	13.12 ± 0.47 ^a^
Total	100.00	100.00	100.00

Data are given as mean ± SD values from experiments performed in triplicate. ^(1)^ Mean values with different letters within the same row are significantly different according to Tukey’s multiple range test (*p* < 0.05).

**Table 4:** Fatty acid profile of the different parts of *Wasabia koreana* Nakai.

Fatty Acid	Composition (%Weight)		
	Leaf	Petiole	Rhizome
Palmitic acid	11.68 ± 0.01 ^c(1)^	17.56 ± 0.02 ^b^	19.37 ± 0.05 ^a^
Stearic acid	3.68 ± 0.01 ^a^	2.25 ± 0.03 ^c^	3.04 ± 0.09 ^b^
Oleic acid (ω-9)	16.33 ± 0.09 ^a^	14.39 ± 0.04 ^b^	14.22 ± 0.13 ^b^
Linoleic acid (ω-6)	8.12 ± 0.07 ^c^	12.19 ± 0.10 ^b^	18.19 ± 0.12 ^a^
Linolenic acid (ω-3)	59.57 ± 0.12 ^a^	52.87 ± 0.14 ^b^	44.25 ± 0.23 ^a^
Arachidic acid	0.26 ± 0.01 ^b^	0.27 ± 0.01 ^b^	0.49 ± 0.04 ^a^
Gondoic acid (ω-9)	0.20 ± 0.02 ^b^	0.27 ± 0.03 ^a^	0.26 ± 0.02 ^a^
Behenic acid	0.15 ± 0.02 ^b^	0.21 ± 0.02 ^a^	0.19 ± 0.09 ^a^
%Saturated fatty acids	15.78 ± 0.21 ^c^	20.29 ± 0.14 ^b^	23.09 ± 0.16 ^a^
%Monounsaturated fatty acids	16.53 ± 0.15 ^a^	14.66 ± 0.09 ^b^	14.48 ± 0.13 ^b^
%Polyunsaturated fatty acids	67.99 ± 0.25 ^a^	65.06 ± 0.31 ^b^	62.44 ± 0.24 ^c^

Data are given as mean ± SD values from experiments performed in triplicate. ^(1)^ Mean values with different letters within the same row are significantly different according to Tukey’s multiple range test (*p* < 0.05).

**Table 5:** Mineral composition of the different parts of *Wasabia koreana* Nakai.

Mineral	Composition (%)		
	Leaf	Petiole	Rhizome
P (phosphorus)	9.11 ± 0.87 ^a(^^1)^	5.34 ± 0.56 ^b^	6.75 ± 0.69 ^b^
S (Sulfur)	20.44 ± 1.97 ^a^	6.89 ± 0.74 ^c^	14.92 ± 1.22 ^b^
K (potassium)	43.75 ± 4.35 ^c^	65.02 ± 4.21 ^a^	57.93 ± 3.23 ^b^
Ca (calcium)	19.66 ± 1.21 ^a^	18.99 ± 2.09 ^a^	16.15 ± 1.43 ^b^
Mg (magnesium)	6.61 ± 0.43 ^a^	3.24 ± 0.33 ^b^	4.02 ± 0.29 ^b^
Fe (iron)	0.15 ± 0.04 ^a^	0.08 ± 0.02 ^b^	0.06 ± 0.02 ^b^
As (arsenic)	0.05 ± 0.03 ^a^	0.06 ± 0.03 ^a^	0.04 ± 0.03 ^a^
Zn (zinc)	0.08 ± 0.02 ^a^	0.04 ± 0.01 ^b^	0.08 ± 0.03 ^a^
Mn (manganese)	0.15 ± 0.02 ^a^	0.14 ± 0.03 ^a^	0.04 ± 0.02 ^b^
Al (aluminum)	0.01 ± 0.00 ^b^	0.19 ± 0.03 ^a^	0.01 ± 0.00 ^b^

Data are given as mean ± SD values from experiments performed in triplicate. ^(1)^ Mean values with different letters within the same row are significantly different according to Tukey’s multiple range test (*p* < 0.05).

**Table 6:** Vitamin C, total phenolic content, and antioxidant capacity of the different parts of *Wasabia koreana* Nakai.

	*Wasabia koreana* Nakai		
	Leaf	Petiole	Rhizome
Vitamin C (mg/g)	108.09 ± 7.43 ^a^	64.72 ± 4.21 ^c^	94.17 ± 6.97 ^b^
Total phenolic content (mg GAE/100 g)	12.75 ± 1.70 ^a(1)^	0.42 ± 0.81 ^b^	3.82 ± 0.60 ^b^
IC_50_ (mg)	7.64 ± 0.54 ^b^	17.24 ± 1.17 ^a^	16.95 ± 0.61 ^a^

Data are given as mean ± SD values from experiments performed in triplicate. ^(1)^ Mean values with different letters within the same row are significantly different according to Tukey’s multiple range test (*p* < 0.05).

**Table 7:** Sinigrin and allyl isothiocyanate (AITC) contents in the different parts of *Wasabia koreana* Nakai.

	*Wasabia koreana* Nakai		
	Leaf	Petiole	Rhizome
Sinigrin (mg/g)	23.45 ± 0.33 ^b(1)^	22.80 ± 0.41 ^b^	92.79 ± 1.81 ^a^
AITC (mg/g)	0.25 ± 0.01 ^b^	0.17 ± 0.02 ^c^	0.89 ± 0.01 ^a^

Data are given as mean ± SD values from experiments performed in triplicate. ^(1)^ Mean values with different letters within the same row are significantly different according to Tukey’s multiple range test (*p* < 0.05).

**Table 8:** Volatile compounds in the different parts of *Wasabia koreana* Nakai.

Compounds	RT (min) ^a^	RI ^b^	Content (μg/g)			Odor Intensity	Odor Description
			Leaf	Petiole	Rhizome		
*Acids*							
2-Hydroxybenzene methanol	5.43	<800	0.34 ± 0.49	n.d. ^c^	n.d.		
Dimethyl silanediol	5.81	<800	n.d.	7.71 ± 10.90	n.d.		
Trifluorolactic acid	18.81	1144	n.d.	0.08 ± 0.11	n.d.		
3-Methyl-2-phenyl pentanoic acid	27.26	1452	0.10 ± 0.14	n.d.	n.d.		
2-Tridecyl ester methoxy acetic acid	27.90	1481	0.69 ± 0.98	n.d.	n.d.		
*Alcohols*							
1,8-Cineole	16.29	1061	1.81 ± 2.55	n.d.	n.d.		
2,5-Dimethyl-2,5-hexanediol	16.42	1065	n.d.	2.02 ± 2.86	n.d.		
*Aldehydes*							
2-1-Phenanthrylbenzaldehyde	14.58	1005	0.27 ± 0.39	n.d.	n.d.		
2-Hexenal	19.26	1159	n.d.	0.08 ± 0.11	n.d.		
3-Hexene-1,6-dialdehyde	24.82	1361	1.17 ± 1.65	9.42 ± 3.32	n.d.		
*Hydrocarbons*							
Ethyl-1,3-dithioisoindoline	8.51	827	5.71 ± 8.08	0.64 ± 0.90	n.d.		
2-Methyl-5-dibenzazepine	9.52	858	0.64 ± 0.91	n.d.	4.47 ± 6.32		
1-Heptadecene	19.10	1154	0.05 ± 0.07	n.d.	n.d.		
Azulene	20.13	1187	n.d.	1.22 ± 1.73	n.d.	1	Wasabi
Nonadecane	25.74	1396	n.d.	1.08 ± 1.52	n.d.		
Tridecane	26.39	1411	0.86 ± 1.22	n.d.	n.d.		
*Heterocyclic*							
2-4′-Nitro-2′-thienylpyrimidine	8.65	832	n.d.	1.73 ± 2.45	n.d.		
Isopropyl isothiocyanate	9.91	869	n.d.	n.d.	1.37 ± 1.93		
3-Methylisothiazole	11.11	901	4.93 ± 1.32	2.84 ± 4.01	8.67 ± 2.26		
Allylisothiocyanate	11.50	914	71.51 ± 8.14	8.99 ± 2.71	117.80 ± 66.60	2	Wasabi
Diethyl-1-propanamine	12.25	938	0.99 ± 1.39	n.d.	n.d.		
4-Isothiocyanato-1-butene	13.94	987	3.01 ± 1.74	n.d.	3.59 ± 5.08		
Dihydro-5,5-dimethyl-2-furanone	14.12	991	n.d.	2.51 ± 3.55	n.d.		
2-Ethyl-3-methyl-4-phenyl-6-chloroquinoline	15.36	1032	0.32 ± 0.45	n.d.	n.d.		
Paromomycin	18.84	1145	n.d.	n.d.	0.36 ± 0.52		
2-Chloro-3,4-diphenylbenzofluoro pyridine	20.26	1192	n.d.	0.09 ± 0.13	n.d.		
3′,4′,5,5′,7-Pentamethoxyflavone	21.19	1226	n.d.	0.39 ± 0.55	n.d.		
Tetramethylpyrazine	22.50	1273	0.09 ± 0.13	n.d.	n.d.		
*Ketone*							
1,7,7-Trimethylbicyclo-2.2.1-heptan-2-one	19.75	1175	46.63 ± 5.95	n.d.	n.d.		

Data values are given as mean ± SD from experiments performed in triplicate. ^a^ RT: retention time, ^b^ RI: retention index, ^c^ n.d.: not detected.

**Table 9:** Volatile compounds in *Wasabia koreana* Nakai identified using an electronic nose. (peak area × 10^3^).

No.	Compounds	Sensory Descriptors	RT (min) ^a^	RI ^b^	Leaf	Petiole	Rhizome
1	Hexadecafluoroheptane	-	10.63	363	2.33 ± 0.16	3.05 ± 0.12	104.96 ± 13.9
2	Ethanol	alcoholic, pungent, sweet	13.18	427	2.15 ± 0.29	1.65 ± 0.08	479.39 ± 34.03
3	2-Propanol	alcoholic	15.85	492	10.25 ± 7.8	11.15 ± 0.85	231.70 ± 11.30
4	Ethyl 2-methylbutyrate	apple, green, sweet	68.89	839	7.02 ± 1.12	22.69 ± 0.81	428.39 ± 58.59
5	Allylisothiocyanate	garlic, pungent, sulfurous	85.90	876	345.78 ± 48.90	1309.28 ± 160.62	13,426.24 ± 653.00
6	Dimethyltrisulfide	onion, rotten food	100.21	955	n.d. ^c^	n.d. ^c^	950.91 ± 99.54
7	Amylpropanoate	apricot, fruity, very sweet	103.04	971	2.14 ± 0.25	1.6 ± 0.34	235.05 ± 24.21
8	1-Octen-3-ol	garlic, herbaceous, spicy	108.39	994	44.00 ± 6.20	105.01 ± 19.11	262.59 ± 32.82
9	Linalool	green, muscat, parsley	120.29	1,096	5.59 ± 2.11	1.5 ± 0.13	597.26 ± 64.23
10	Decanal	green, herbaceous, peel	131.60	1,206	0.53 ± 0.21	0.42 ± 0.03	74.82 ± 9.47

Data are given as mean ± SD values from experiments performed in triplicate. ^a^ RT: retention time; ^b^ RI: retention index, ^c^ n.d.: not detected.
